# Fabrication and Properties of Zn-Containing Intermetallic Compounds as Sacrificial Anodes of Zn-Based Implants

**DOI:** 10.3390/ma18092057

**Published:** 2025-04-30

**Authors:** Kelei Li, Junwei Li, Tiebao Wang, Xin Wang, Yumin Qi, Lichen Zhao, Chunxiang Cui

**Affiliations:** Hebei Key Laboratory of New Functional Materials, School of Materials Science and Engineering, Hebei University of Technology, Tianjin 300400, China

**Keywords:** Zn-based implants, sacrificial anodes, MgZn_2_, CaZn_13_, Ca_2_Mg_6_Zn_3_

## Abstract

In the field of degradable metals, Zn-based implants have gradually gained more attention. However, the relatively slow degradation rate compared with the healing rate of the damaged bone tissue, along with the excessive Zn^2+^ release during the degradation process, limit the application of Zn-based implants. The use of intermetallic compounds with more negative electrode potentials as sacrificial anodes of Zn-based implants is likely to be a feasible approach to resolve this contradiction. In this work, three intermetallic compounds, MgZn_2_, CaZn_13_, and Ca_2_Mg_6_Zn_3_, were prepared. The phase structures, microstructures, and relevant properties, such as thermal stability, in vitro degradation properties, and cytotoxicity of the compounds, were investigated. The XRD patterns indicate that the MgZn_2_ and CaZn_13_ specimens contain single-phase MgZn_2_ and CaZn_13_, respectively, while the Ca_2_Mg_6_Zn_3_ specimen contains Mg_2_Ca and Ca_2_Mg_6_Zn_3_ phases. After purifying treatment in 0.9% NaCl solution, high purity Ca_2_Mg_6_Zn_3_ phase was obtained. Thermal stability tests suggest that the MgZn_2_ and CaZn_13_ specimens possess good thermal stability below 773 K. However, the Ca_2_Mg_6_Zn_3_ specimen melted at around 739.1 K. Polarization curve tests show that the corrosion potentials of MgZn_2_, CaZn_13_, and Ca_2_Mg_6_Zn_3_ in simulated body fluid (SBF) were −1.063 V_SCE_, −1.289 V_SCE_, and −1.432 V_SCE_, which were all more negative than that of the pure Zn specimen (−1.003 V_SCE_). Clearly, these compounds can act as sacrificial anodes in Zn-based implants. The immersion tests indicate that these compounds were degraded according to the atomic ratio of the elements in each compound. Besides that, the compounds can efficiently induce Ca-P deposition in SBF. Cytotoxicity tests demonstrate that the 10% extracts prepared from these compounds exhibit good cell activity on MC3T3-E1 cells.

## 1. Introduction

As biodegradable implants temporarily present in the body, Zn and its alloys have become a research hotspot in the field of degradable metals in recent years due to their acceptable biocompatibility and more suitable degradation rate than Mg-based and Fe-based materials [1,2,3,4,5,6,7,8,9,10,11,12,13,14]. However, recent studies have shown that the degradation rate of Zn-based materials is relatively slow in orthopedic applications, which inevitably leads to long-term retention of implants in the body and resulting metabolic complications [15,16]. Since the degradation rate of implants should match the bone regeneration rate as much as possible, the implants must maintain their mechanical integrity in the body for approximately 3–6 months and fully degrade within 1–2 years [15,16,17,18]. To meet the requirements, Refs. [19,20] reported that the degradation rate of implants should be about 0.2 mm/year. Refs. [16,17] reported that the degradation rate should be 0.5 mm/year (plates and screws). Shuai et al. [21] believed that 0.2–0.5 mm/year should be an appropriate degradation rate, which may be due to the fact that the healing periods or the bone regeneration periods are different for the specific bone tissues [16,20,22]. Zhao et al. [23] have summarized the degradation rates of 130 Zn-based specimens (pure Zn and various Zn alloys) after immersion in corrosive solutions for different periods. The results showed that the degradation rates of 120 Zn-based specimens were less than 0.2 mm/year. Among the remaining 10 specimens whose degradation rates were greater than 0.2 mm/year, the maximum rate of these specimens was only about 0.253 mm/year. Not only that, Zhao et al. [23] also reported that 10 porous Zn-based scaffolds among the total 25 scaffold specimens also exhibited a degradation rate of less than 0.2 mm/year during the immersion tests [23]. In addition, it also should be noted that the degradation rate of degradable metals in vivo may be slower than that in vitro due to the essential differences between the in vivo and in vitro environments [24]. For example, Witte et al. [25] reported that the degradation rates of AZ91D and LAE442 alloys in vitro (electrochemical tests) were approximately 10^3^–10^4^ times higher than that those measured in vivo. Ren et al. [26] also reported that the porous Zn-Cu scaffolds exhibited a slower degradation rate in vivo than in vitro. In addition to the relatively slow degradation rates, the second drawback of Zn-based implants is the excessive release of Zn^2+^ during the degradation process. It is known that Zn is only a trace element in the human body [27], and the tolerance threshold of cells and tissues to Zn^2+^ is rather low. As a result, the excessive release of Zn^2+^ can easily cause severe cytotoxicity in vitro and delayed osseointegration in vivo [16,28]. For example, Yang et al. [28] reported that the cell viability of MC3T3-E1 cells after culturing in the extract prepared from a pure Zn specimen for 1–4 days was only about 15.8–51.4%. After implantation of the pure Zn specimen into a Sprague-Dawley rat for 4 weeks, a fibrous connective tissue layer was found around the implant, and only limited sites of direct attachment of new bone to the implant were observed [28]. When Zn-based materials were prepared into porous scaffolds, the porous scaffolds would exhibit faster degradation rates and greater Zn^2+^ release, which then resulted in lower cell viability [3,29,30]. Based on the above statements, solving the contradiction between the relatively slow degradation rate and the excessive release of Zn^2+^ during the degradation process naturally becomes an important research direction in the field of degradable Zn-based implant materials.

To resolve the contradiction, introducing sacrificial anodes with electrode potentials more negative than Zn into Zn-based implants should be a viable approach. Yang et al. [28] prepared Zn-*x*Mg composites (*x* = 1, 2, 5 wt.%) using pure Zn powders and pure Mg powders as raw materials by the spark plasma sintering method. Their experimental results showed that the Mg-rich particles with a core–shell structure (the inner core was MgZn_2_ phase, and the outer shell was Mg_2_Zn_11_ phase) were uniformly distributed in the composites. Electrochemical tests indicated that the composites had more negative corrosion potentials and faster degradation rates than the pure Zn specimen. Furthermore, the Mg-rich phases in the composites indeed degraded preferentially, forming obvious corrosion pits. The results of cytotoxicity tests demonstrated that the Zn^2+^ concentrations in the extracts of Zn-*x*Mg composites were lower than that in the extract of the pure Zn specimen, and the composites also exhibited better cell viability than the pure Zn specimen. The in vivo implantation experiments conducted in Sprague-Dawley rats also showed that more sites on the Zn-5Mg composite could directly bond to new bone after 4 weeks of implantation, indicating an improvement in bone integration ability compared with the pure Zn specimen.

Based on the experimental results of Yang et al. [28], it can be confirmed that the compounds MgZn_2_ and Mg_2_Zn_11_ can be used as sacrificial anodes in Zn-based implants. However, studies on the degradation properties and degradation mechanisms of MgZn_2_, Mg_2_Zn_11_, and other compounds that can also be used as sacrificial anodes are very limited. Currently, only very few studies have reported the corrosion potentials of MgZn_2_ and Mg_2_Zn_11_ in 3.5% NaCl solution [31,32], as well as the pH variation in the Hank’s solution soaking of a CaZn_2_ specimen [33]. Apparently, the current information on the degradation properties and degradation mechanisms of the compounds used as sacrificial anodes is not yet sufficient to develop biodegradable Zn-based implants with suitable and controllable degradation rates and good biocompatibility.

In this work, three intermetallic compounds, MgZn_2_, CaZn_13_, and Ca_2_Mg_6_Zn_3_, were prepared, and their phase structures and microstructures were characterized. The thermal properties, degradation behaviors, as well as the cytotoxicity of these compounds were also investigated. The experimental results are expected to provide assistance for the preparation of biodegradable Zn-based composites containing these compounds in the next step, and also provide useful help in clarifying the mechanism by which these compounds regulate the degradation behavior and cytotoxicity of Zn-based composites.

## 2. Materials and Methods

### 2.1. Preparation of the Intermetallic Compounds

Commercially pure Zn (≥99.995%), pure Mg (≥99.95%), and pure Ca (≥99.0%) ingots were used as raw materials. Three intermetallic compounds having nominal compositions of MgZn_2_, CaZn_13_, and Ca_2_Mg_6_Zn_3_ were prepared by a vacuum induction melting furnace under argon protection. Then, the obtained MgZn_2_, CaZn_13_, and Ca_2_Mg_6_Zn_3_ ingots were annealed at 500 °C, 470 °C, and 320 °C for 15 h under argon atmosphere, respectively. After that, the oxide layers on the specimens were removed. Due to the report in Ref [34] that when Ca, Mg, and Zn were melted in an atomic ratio of Ca/Mg/Zn = 1/3/1.5 to prepare Ca_2_Mg_6_Zn_3_, the resulting product was likely to contain Mg_2_Ca phase, and the annealed Ca_2_Mg_6_Zn_3_ ingot still needed to be purified. The annealed Ca_2_Mg_6_Zn_3_ ingot was then immersed in 0.9% NaCl solution until the specimen completely disintegrated into powders. The powders were collected and pressurized into a cylinder with a diameter of 20 mm at 400 MPa for 3 min. After that, the cylinder compact was sintered at 390 °C for 1.5 h under the protection of argon atmosphere. Hereafter, unless otherwise specified, the intermetallic compounds used for phase structure, microstructure, and property characterizations are in an annealed state for the MgZn_2_ and CaZn_13_ specimens, while the Ca_2_Mg_6_Zn_3_ specimen is in a sintered state.

### 2.2. Phase Structure and Microstructure Characterizations of the Intermetallic Compounds

The phase structures of the obtained compounds were identified by an X-ray diffractometer (SmartLab, Rigaku, Tokyo, Japan) with Cu K_α_ radiation (2°/min). The microstructures of the specimens were characterized by a scanning electron microscope (SEM, S-4800 or SU-3800, Hitachi, Tokyo, Japan). An energy-dispersive X-ray spectrometer (EDS) equipped on the scanning electron microscope was used to determine the chemical compositions of specimens. Before observing the microstructures, the specimens were ground, polished, and then etched with a 4% nitric acid–alcohol solution.

### 2.3. Microhardness Tests of the Intermetallic Compounds

The microhardness of the intermetallic compounds was measured by a Vickers hardness tester (HVS-30Z, Shanghai Aolong Xingdi Testing Equipment Co., LTD., Shanghai, China). The applied load and the holding time were 196.1 N and 15 s, respectively.

### 2.4. Thermal Stability Tests of the Intermetallic Compounds

Thermal stability tests of the intermetallic compounds were carried out on a Themys ONE DSC/TG simultaneous analyzer (Setaram Instruments, Lyon, France). The specimens having a weight of 20–30 mg were loaded into alumina crucibles, and then they were heated from room temperature to 773 K at a heating rate of 5 K/min under nitrogen atmosphere to measure the differential scanning calorimetry (DSC) curves and the thermalgravimetric (TG) curves.

### 2.5. Electrochemical Tests of the Intermetallic Compounds

Polarization curves of the intermetallic compounds were measured by an electrochemical workstation (CHI660-E, Shanghai, China). The intermetallic compound specimens were used as the working electrodes, a saturated calomel electrode (SCE) served as the reference electrode, and a graphite rod acted as the counter electrode. The used corrosive media were 0.9% NaCl solution and simulated body fluid (SBF). The chemical composition of the SBF is listed in Table 1. The pH value of the SBF (37 °C) was adjusted to 7.4 using tris(hydroxymethyl) aminomethane and 1 M HCl solution. During the whole tests, the corrosive media were kept at 37 °C. Before measuring the polarization curves, the working electrodes were immersed in the corrosive media for 90 min to obtain a stable open circuit potential. After that, the polarization curves of the specimens were measured at a scanning rate of 0.5 mV/s. For comparison, the polarization curve of an as-cast pure Zn specimen was also tested.

### 2.6. Immersion Tests of the Intermetallic Compounds

The immersion tests of the intermetallic compounds were performed in 0.9% NaCl solution and SBF solution, respectively.

To carry out NaCl solution immersion tests, the pH values of the used NaCl solutions were firstly adjusted to 4.0 using a 1 M HCl solution. The intermetallic compound specimens (~1 g) were then immersed in the solutions (150 mL) for 24 h at room temperature. During immersion tests, the immersion solutions were continuously stirred magnetically. At the end of the immersion tests, the metal ion concentrations in the solutions were measured by an inductively coupled plasma-optical emission spectrometer (Agilent 5100, Santa Clara, CA, USA).

SBF immersion tests were performed in 150 mL of SBF solutions at 37 °C for different periods, and the exposed area of the compound specimens was around 1 cm^2^. During the immersion tests, the pH values of the solutions were measured. After immersion tests, the specimens were taken out from the immersion solutions, and rinsed with deionized water. After drying naturally, the corrosion products deposited on the specimens were characterized by a scanning electron microscope (S-4800, Hitachi) and a Fourier transform infrared spectrometer (FTIR, Vertex 80 V, Bruker, Billerica, MA, USA). Then, the corrosion products were removed with a CrO_3_ solution (200 g/L), and the corrosive morphologies of the specimens were also observed.

### 2.7. Cytotoxicity Tests of the Intermetallic Compounds

The cytotoxicity of the intermetallic compounds was evaluated by an indirect contact method, and the cells for tests were MC3T3-E1 cells. The used specimens were 100–150 μm intermetallic compound powders, and the cell culture medium was α-minimum essential medium (saibaikang, iCell-0003, Shanghai, China) containing 10% fetal bovine serum (Procell, 164210-50, Wuhan, China). Detailed experimental steps can be found in Refs. [3,36]. It should be noted that the metal ion concentrations in the 100% extracts prepared by incubating the sterilized specimens in the cell culture medium for 24 h were also measured by an inductively coupled plasma-optical emission spectrometer (Agilent 5110). The cellular activity (i.e., relative growth rate, RGR) of the specimens after incubation in the extracts with different concentrations for different periods was determined by the equation provided in Ref. [3].

## 3. Results and Discussion

### 3.1. Phase Structures of the Intermetallic Compounds

**Figure 1:**
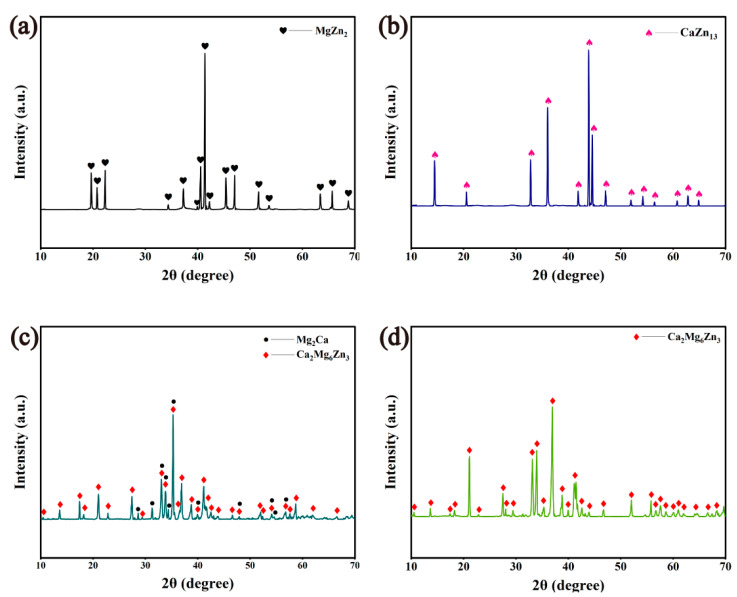
X-ray diffraction patterns of the annealed (**a**) MgZn_2_, (**b**) CaZn_13_, (**c**) Ca_2_Mg_6_Zn_3_ specimens, and (**d**) the sintered Ca_2_Mg_6_Zn_3_ specimen.

Figure 1 depicts the X-ray diffraction patterns of the MgZn_2_, CaZn_13_, and Ca_2_Mg_6_Zn_3_ specimens. It can be seen that only single-phase MgZn_2_ (PDF card number: 04-008-7744) and CaZn_13_ (PDF card number: 97-018-4414) were identified in the annealed MgZn_2_ and CaZn_13_ specimens, respectively, while no other phases were found. The results suggest that both the prepared MgZn_2_ and CaZn_13_ specimens, after annealing, exhibit high purity. However, Mg_2_Ca phase (PDF card number: 03-065-3583) was also detected in the annealed Ca_2_Mg_6_Zn_3_ specimen in addition to Ca_2_Mg_6_Zn_3_ phase (PDF card number: 00-012-0266) (Figure 1c). When the annealed Ca_2_Mg_6_Zn_3_ specimen was disintegrated into powders in NaCl solution and then these powders were compacted and sintered, the obtained specimen only contained Ca_2_Mg_6_Zn_3_ phase (Figure 1d). The reason why Mg_2_Ca phase could be removed from the Ca_2_Mg_6_Zn_3_ specimen during immersion in NaCl solution should be attributed to the preferential degradation of the Mg_2_Ca phase. It is known that the standard electrode potentials of Ca = Ca^2+^ + 2e^−^, Mg = Mg^2+^ + 2e^−^, and Zn = Zn^2+^ + 2e^−^ are −2.87 V_SHE_, −2.372 V_SHE_, and −0.762 V_SHE_, respectively. Therefore, it can be inferred that the electrode potential of Mg_2_Ca, which is composed of Mg and Ca elements with more negative electrode potentials, is lower than that of Ca_2_Mg_6_Zn_3_, just as the electrode potentials of MgZn_2_ and Mg_2_Zn_11_ are lower than that of pure Zn reported in Refs. [31,32]. When the annealed Ca_2_Mg_6_Zn_3_ specimen was immersed in NaCl solution, numerous corrosion couples formed between the Mg_2_Ca phase and the Ca_2_Mg_6_Zn_3_ phase. Clearly, the Mg_2_Ca phase that had a more negative electrode potential would preferentially degrade as a sacrificial anode. When the Mg_2_Ca phase completely degraded, the residual phase was just Ca_2_Mg_6_Zn_3_.

### 3.2. Microstructures of the Intermetallic Compounds

**Figure 2:**
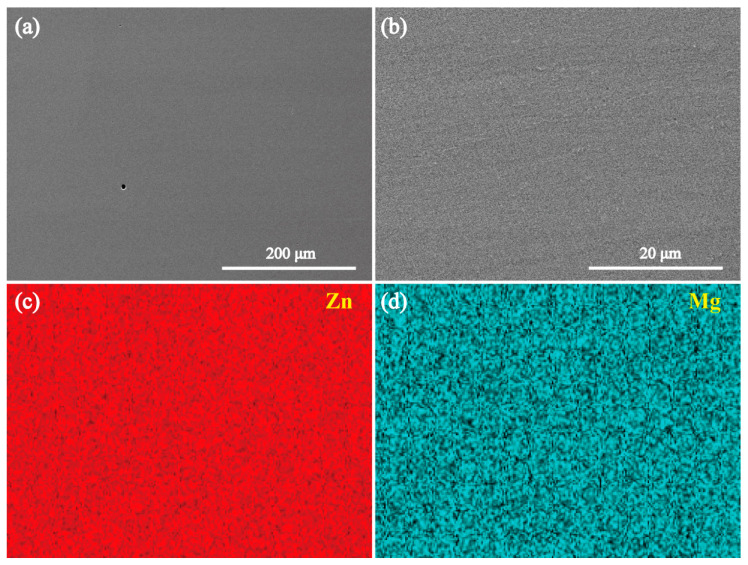
(**a**,**b**) SEM images of the MgZn_2_ specimen, and (**b**) a high magnification image of a local area in (**a**). (**c**,**d**) The elemental mapping results for the area in (**b**).

**Figure 3:**
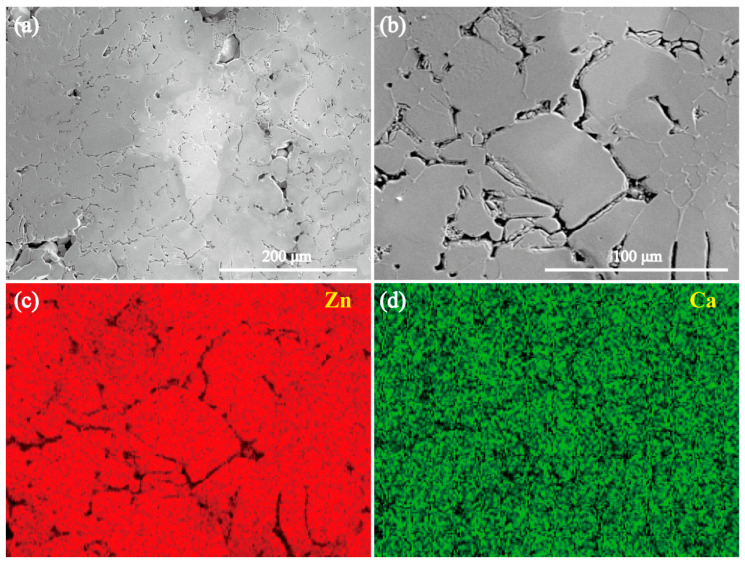
(**a**,**b**) SEM images of the CaZn_13_ specimen, and (**b**) a high magnification image of a local area in (**a**). (**c**,**d**) The elemental mapping results for the area in (**b**).

**Figure 4:**
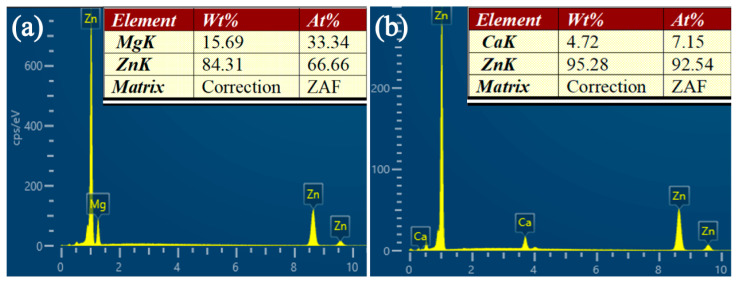
(**a**) The EDS results of the area shown in Figure 2b, (**b**) the EDS results of the area shown in Figure 3b.

Figure 2 and Figure 3 present the SEM images and elemental mapping results of the MgZn_2_ and CaZn_13_ specimens, respectively. The EDS results of the areas shown in Figure 2b and Figure 3b are presented in Figure 4a and 4b, respectively. As seen in Figure 2, the contrast of the MgZn_2_ specimen is almost uniform; no grains and grain boundaries were observed. The elemental mapping results show that the Zn and Mg elements on the specimen are uniformly distributed (Figure 2c,d). Not only that, the EDS result (Figure 4a) for the area shown in Figure 2b also suggests that the atomic ratio of Mg/Zn is 1/2.00, indicating that the compound is MgZn_2_. Clearly, the result is consistent with the XRD result presented in Figure 1a. It should be noted that the microstructure shown in Figure 2 is quite different from that of the MgZn_2_ specimen reported by Yao et al. [31]. In the microstructure of MgZn_2_ reported by Yao et al. [31], MgZn_2_ grains are separated by obvious grain boundaries. For the CaZn_13_ specimen, the contrast of the specimen surface is also almost uniform except for the grain boundaries. The elemental mapping results imply that the distributions of Zn and Ca elements on the specimen are almost uniform except for the grain boundaries (Figure 3c,d). The EDS result (Figure 4b) for the area shown in Figure 3b suggests that the atomic ratio of Ca/Zn is 1/12.94, suggesting that the compound is CaZn_13_. Clearly, the result is also consistent with the XRD result presented in Figure 1b.

**Figure 5:**
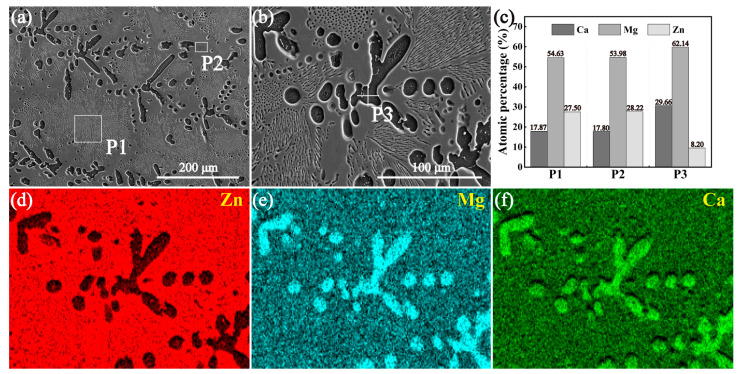
(**a**,**b**) SEM images of the annealed Ca_2_Mg_6_Zn_3_ specimen, and (**b**) a high magnification image of a local area in (**a**). (**c**) The EDS results of P1, P2 in (**a**) and P3 in (**b**). (**d**–**f**) The elemental mapping results for the area in (**b**).

**Figure 6:**
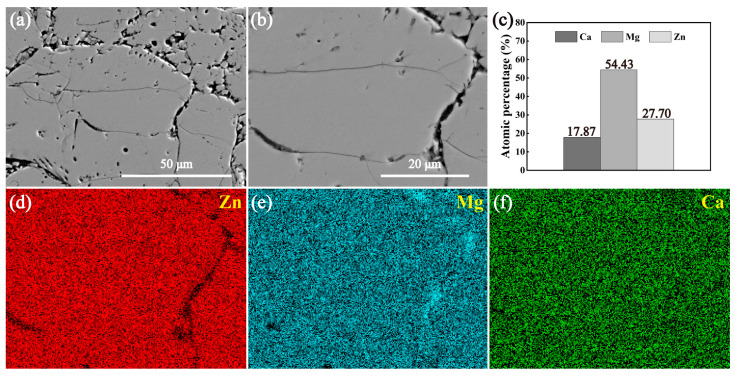
(**a**,**b**) SEM images of the sintered Ca_2_Mg_6_Zn_3_ specimen, and (**b**) a high magnification image of a local area in (**a**). (**c**) The EDS results for the area shown in (**b**). (**d**–**f**) The elemental mapping results for the area in (**b**).

Figure 5 and Figure 6 show the SEM images and EDS mapping results of the annealed and the sintered Ca_2_Mg_6_Zn_3_ specimens, respectively. For the annealed Ca_2_Mg_6_Zn_3_ specimen, it can be seen that concave dark-colored phase and convex, relatively light-colored phase exist in the specimen (Figure 5a,b). The elemental mapping results suggest the concave dark-colored phase is rich in Mg and Ca, but poor in Zn element (Figure 5d–f). However, the convex, relatively light-colored phase is rich in Zn element, but relatively poor in Mg and Ca elements (Figure 5d–f). The EDS results of the rectangular areas P1 and P2 shown in Figure 5a suggest that the atomic ratios of Ca/Mg/Zn are 1/3.06/1.43 and 1/3.03/1.59, respectively (Figure 5c). Clearly, the two ratios are close to the atomic ratio of Ca, Mg, and Zn elements in Ca_2_Mg_6_Zn_3_. The atomic ratio of Ca/Mg/Zn at point P3 in Figure 5b is 1/2.10/0.28 (Figure 5c). Combined with the XRD pattern shown in Figure 1c, it can be determined that the concave dark-colored phase is Mg_2_Ca, and the convex, relatively light-colored phase is Ca_2_Mg_6_Zn_3_. In addition, the Mg_2_Ca phase appears concave after the Ca_2_Mg_6_Zn_3_ specimen is corroded by the metallographic etching solution, indicating that the Mg_2_Ca phase is more susceptible to corrosion than the Ca_2_Mg_6_Zn_3_ phase. This result is most likely related to the preferential degradation of the Mg_2_Ca phase as a sacrificial anode. For the sintered Ca_2_Mg_6_Zn_3_ specimen, it can be seen that the concave dark-colored phase existing in Figure 5 is no longer present (Figure 6a,b). The crevices in the specimen should be attributed to the fact that the green compact itself used for sintering was not completely dense and that the powders in the compact did not fully achieve metallurgical bonding during the sintering process. The elemental mapping results shown in Figure 6d–f suggest that the distributions of Zn, Mg, and Ca elements are uniform. The EDS result for the area shown in Figure 6b implies that the atomic ratio of Ca/Mg/Zn is 1:3.05:1.55 (Figure 6c), which indicates that the sintered specimen should be Ca_2_Mg_6_Zn_3_. Clearly, the result is still consistent with the XRD result depicted in Figure 1d.

### 3.3. Microhardness of the Intermetallic Compounds

**Figure 7:**
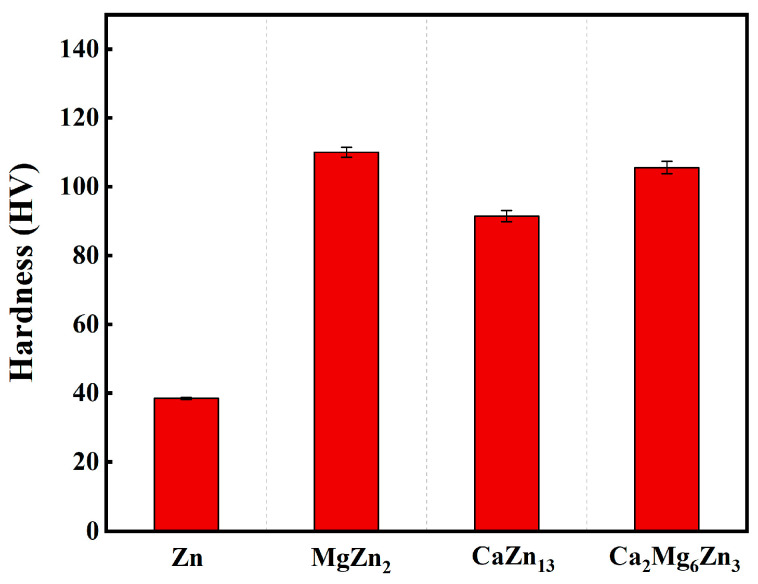
Microhardness results of an as-cast pure Zn specimen and the intermetallic compounds.

The microhardness of the MgZn_2_, CaZn_13_, and Ca_2_Mg_6_Zn_3_ specimens is plotted in Figure 7. For comparison, the microhardness of an as-cast pure Zn specimen is also provided in Figure 7. The measured hardness values of the MgZn_2_, CaZn_13_, and Ca_2_Mg_6_Zn_3_ specimens are 111.0 ± 1.5 HV, 94.2 ± 1.6 HV, and 108.6 ± 1.8 HV, respectively. Clearly, all of these compounds are harder than the as-cast pure Zn (38.5 ± 0.3 HV). The result suggests that the Zn-based composites reinforced by these compounds will have better mechanical properties. Furthermore, these intermetallic compounds in Zn matrix composites can also serve as sacrificial anodes to effectively regulate the degradation rates and cytotoxicity of the composites. In this way, these compounds will be able to achieve simultaneous regulation of the structure and function of the composites.

### 3.4. DSC/TG Curves of the Intermetallic Compounds

**Figure 8:**
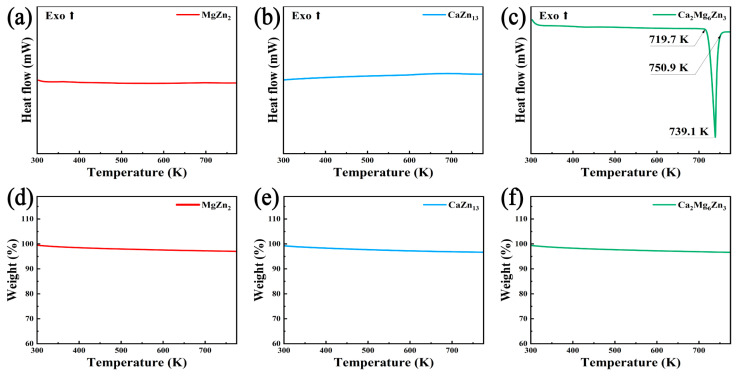
DSC (**a**–**c**) and TG (**d**–**f**) curves of the intermetallic compounds.

Figure 8 presents the DSC/TG curves of the MgZn_2_, CaZn_13_, and Ca_2_Mg_6_Zn_3_ specimens. The DSC curves shown in Figure 8a,b suggest that no endothermic/exothermic peaks appeared throughout the heating of the MgZn_2_ and CaZn_13_ specimens from room temperature to 773 K. The TG curves in Figure 8d,e suggest that the weight losses of the MgZn_2_ and CaZn_13_ specimens are also very slight during the heating processes. Apparently, both the MgZn_2_ and CaZn_13_ phases can exhibit good thermal stability below 773 K. This result can be attributed to the high purity of the specimens and the fact that the thermal analysis temperature did not reach their individual melting points (MgZn_2_: ~863 K [31], CaZn_13_: ~942 K [37]). For the Ca_2_Mg_6_Zn_3_ specimen, there is a clear endothermic peak between 719.7 and 750.9 K, and the peak tip corresponds to a temperature of 739.1 K. Based on the morphology of the Ca_2_Mg_6_Zn_3_ specimen in the alumina crucible after thermal analysis test obviously showing the signs of re-solidification after melting and the literature reported by Wasiur-Rahman et al. [34], it can be inferred that the endothermic peak on the DSC curve was caused by the melting of the Ca_2_Mg_6_Zn_3_ specimen. Although the Ca_2_Mg_6_Zn_3_ specimen melted during the thermal analysis test, it is still feasible to use Ca_2_Mg_6_Zn_3_ powders as sacrificial anodes to prepare Zn-based composites reinforced by the compound using a vacuum hot press sintering method. This is because the hot press sintering temperature of Zn-based composites is generally lower than the melting point of Zn (around 692 K). Clearly, the onset melting temperature of Ca_2_Mg_6_Zn_3_ (719.7 K) is still higher than the sintering temperature. In addition, it is noted that the weight loss shown in Figure 8f is also slight.

### 3.5. Polarization Curves of the Intermetallic Compounds

**Figure 9:**
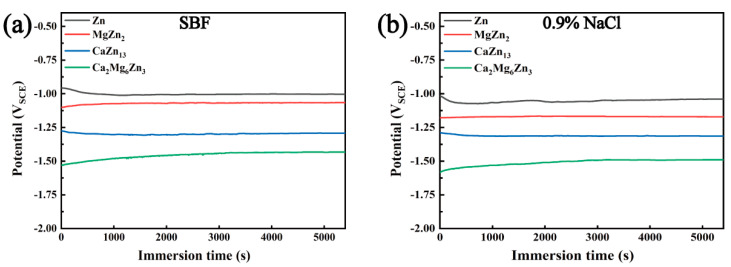
OCP–time curves of the intermetallic compounds and an as-cast pure Zn specimen in (**a**) SBF and (**b**) 0.9% NaCl solution, respectively.

**Figure 10:**
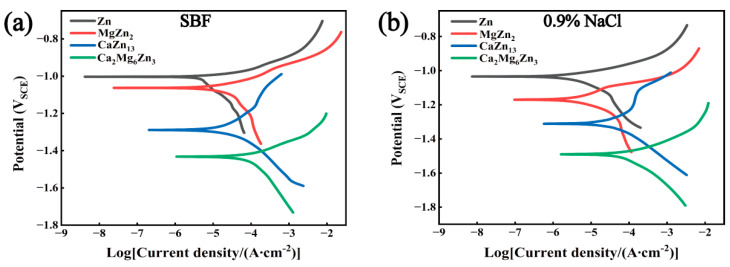
Polarization curves of the intermetallic compounds and an as-cast pure Zn specimen in (**a**) SBF and (**b**) 0.9% NaCl solution, respectively.

Figure 9 presents the open circuit potential (OCP) variation curves with the immersion time of the intermetallic compounds and an as-cast pure Zn specimen in SBF and 0.9% NaCl solution. The measured polarization curves of the specimens in both corrosive solutions are provided in Figure 10. The corrosion potentials (*E*_corr_) and the corrosion current densities (*I*_corr_) derived from the polarization curves by the Tafel extrapolation method are summarized in Table 2. As seen in Figure 9, when the specimens were immersed in SBF or NaCl solutions for 90 min, the OCPs of the specimens were basically stable. The OCP order of the specimens in both solutions is Zn > MgZn_2_ > CaZn_13_ > Ca_2_Mg_6_Zn_3_. The polarization curve measurements suggest that the intermetallic compounds and the pure Zn specimen exhibit a corrosion potential order consistent with the OCP order (Figure 10 and Table 2). The results clearly confirm that the three compounds can act as sacrificial anodes in Zn-based composites to protect the cathodic Zn matrix. The reason why the corrosion potential of these compounds is lower than that of the pure Zn specimen can be attributed to the fact that the electrode potentials for the anodic oxidation reactions of Ca and Mg are significantly lower than that of Zn (−2.87 V_SHE-Ca_ and −2.372 V_SHE-Mg_ vs. −0.762 V_SHE-Zn_). Therefore, the electrode potentials of the compounds formed by the combination of Zn with Mg and/or Ca elements is inevitably lower than that of pure Zn. The inference has been verified by some studies to be reliable. For example, Dong et al. [38] reported that the corrosion potential of MgZn_2_ in SBF was around −1.221 V_SCE_, which was more negative than that of pure Zn (−1.218 V_SCE_). Byun et al. [32] reported that the corrosion potential of MgZn_2_ in 3.5% NaCl solution was −1.083 V_Ag/AgCl_, which was also lower than that of pure Zn (−1.040 V_Ag/AgCl_). The experimental results reported by Yao et al. [31] also suggest that the corrosion potential of MgZn_2_ in 3.5% NaCl is lower than that of pure Zn (−0.79 V_SHE_ vs. −0.74 V_SHE_). Besides that, the results of the electrochemical tests in our work also suggest that the corrosion potential of the compounds is not only related to the electrode potential of non-Zn elements existing in the compounds, but also influenced by the contents of these non-Zn elements. In addition to the corrosion potential, the corrosion current density of the compounds and pure Zn specimen also exhibits the same order in SBF and 0.9% NaCl solution, that is, pure Zn < MgZn_2_ < CaZn_13_ < Ca_2_Mg_6_Zn_3_. Similar results were also reported in the literature. For example, Dong et al. [38] reported that the corrosion current density of MgZn_2_ was also higher than that of pure Zn (47.5 μA/cm^2^ vs. 39.5 μA/cm^2^). The reason for the greater current densities of the compounds may be due to the fact that these compounds possess more negative potentials, thus making them exhibit stronger chemical activity in corrosive media. In addition, the oxide films on these compounds may not be as dense as that on the pure Zn specimen (the oxide densification coefficients of the three oxides are α_CaO_ = 0.647, α_MgO_ = 0.779, and α_ZnO_ = 1.585, respectively).

### 3.6. Degradation Behaviors of the Intermetallic Compounds in Corrosive Solutions

The measured metal ion concentrations in the 0.9% NaCl solutions after soaking the intermetallic compounds for 24 h are listed in Table 3. The calculated concentration ratios of the metal ions dissolved in the NaCl solutions soaking the MgZn_2_, CaZn_13_, and Ca_2_Mg_6_Zn_3_ specimens are 1/1.87 (Mg^2+^/Zn^2+^), 1/13.29 (Ca^2+^/Zn^2+^), and 1/3.07/1.60 (Ca^2+^/Mg^2+^/Zn^2+^), respectively. It can be seen that the concentration ratio of the metal ions dissolved in the NaCl solution approximately corresponds to the atomic ratio of the elements in each compound, that is, these compounds should be degraded according to the atomic ratio of the elements in each compound. Then the anodic degradation reactions of these compounds during immersion tests can be expressed as follows:

MgZn_2_ = Mg^2+^ + 2Zn^2+^ + 6e^−^ (1)

CaZn_13_ = Ca^2+^ + 13Zn^2+^ + 28e^−^ (2)

Ca_2_Mg_6_Zn_3_ = 2Ca^2+^ + 6Mg^2+^ + 3Zn^2+^ + 22e^−^ (3)

**Figure 11:**
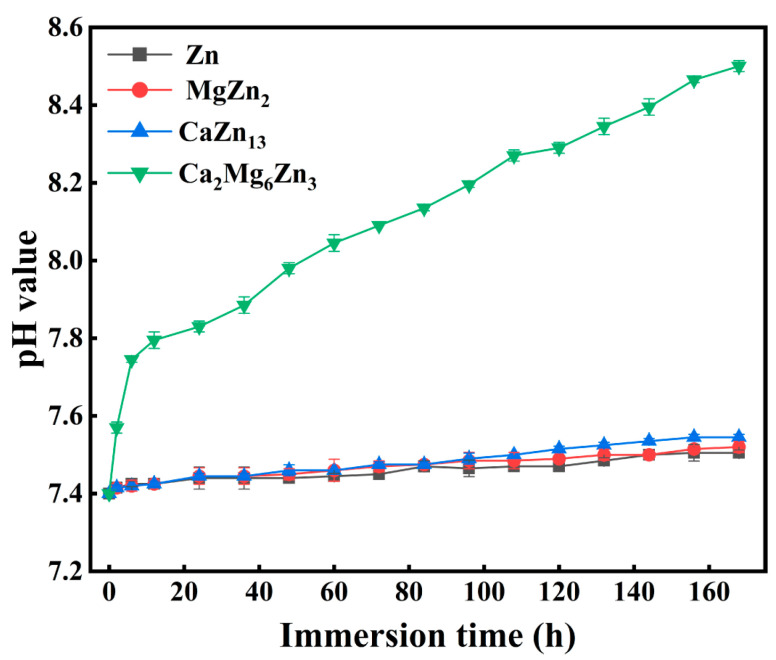
The pH variations of the SBF solutions soaking the intermetallic compounds and pure Zn specimens during immersion tests.

Figure 11 depicts the pH variations of the SBF solutions soaking the intermetallic compounds and as-cast pure Zn specimens during immersion tests. For the as-cast pure Zn, MgZn_2_, and CaZn_13_ specimens, the pH values of the SBF solutions containing these specimens show very similar variations during the initial 24 h of immersion, all increasing from 7.40 to 7.44 or 7.45 with a small slope. Then, the pH values continued to slowly increase with a much smaller slope until the end of the immersion. At last, the pH values of the SBF solutions containing the as-cast pure Zn, MgZn_2_, and CaZn_13_ specimens reached 7.51, 7.52, and 7.55, respectively. In addition, it is also worth noting that the pH value of the SBF containing the CaZn_13_ specimen is slightly higher than that containing the MgZn_2_ specimen, and the pH value of the SBF containing the MgZn_2_ specimen is also slightly higher than that containing the pure Zn specimen in the middle and late stages of the immersion tests (after 72 h of immersion). For the Ca_2_Mg_6_Zn_3_ specimen, the pH variation in the SBF is quite different. After the first 6 h of immersion, the pH value quickly increased from 7.40 to 7.75. Then the pH value of the immersion solution continued to rise slowly with time. At the end of the immersion, the pH of the soaking solution reached 8.5. Obviously, the SBF containing the Ca_2_Mg_6_Zn_3_ specimen exhibited the largest pH growth rate as well as the highest pH value during the entire immersion tests among the four specimens. Since the cathodic reaction associated with the electrochemical corrosion of Zn-based materials is known to release OH^−^ ions (Equation (4)) [3,35], the pH value of the immersion solution can reflect to some extent the degradation rate of the specimen. Based on this point, it can be concluded that the Ca_2_Mg_6_Zn_3_ specimen has the fastest degradation rate, followed by the CaZn_13_ and MgZn_2_ specimens. The as-cast pure Zn specimen exhibits the slowest degradation rate. Apparently, this result is consistent with the degradation rate ordering obtained from the polarization curves (Table 2).

2H_2_O+ O_2_ + 4e^−^ = 4OH^−^ (4)

**Figure 12:**
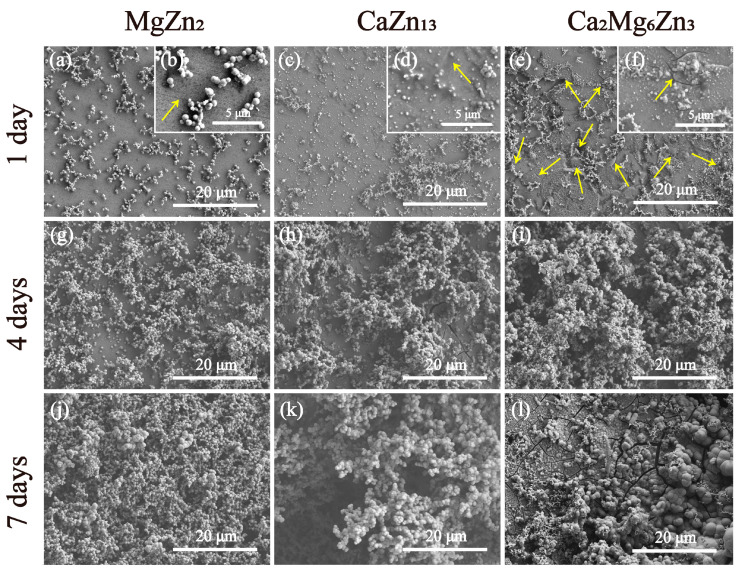
SEM images of the MgZn_2_ (**a**,**b**,**g**,**j**), CaZn_13_ (**c**,**d**,**h**,**k**) and Ca_2_Mg_6_Zn_3_ (**e**,**f**,**i**,**l**) specimens after immersion in SBF for different periods.

Figure 12 shows the SEM images of the intermetallic compounds after immersion in SBF for different periods of time. As shown in Figure 12a,b, after 1 day of the immersion test, some light-colored, nearly spherical corrosion products with varying sizes had deposited on the MaZn_2_ specimen, either in clusters or as individual particles. Apparently, many areas of the MgZn_2_ surface remained exposed at this time, and an original scratch on the specimen’s surface could also be seen (indicated by the yellow arrow in Figure 12b). Similar to the MaZn_2_ specimen, both clustered and individual granular corrosion products were observed on the CaZn_13_ and Ca_2_Mg_6_Zn_3_ specimens, but the amount of corrosion products increased (Figure 12c–f). Additionally, a tiny crack was found in the dark-colored area of the CaZn_13_ specimen (indicated by the yellow arrow in Figure 12d). Yuan et al. [36] and Zhang et al. [3] believed that the formation of cracks was caused by the dehydration of the corrosion products during the drying of the specimen after immersion tests. The finer the crack, the thinner the layer of corrosion products is likely to be. For the Ca_2_Mg_6_Zn_3_ specimen, a greater number of cracks were observed on the specimen surface (indicated by the yellow arrows in Figure 12e,f), and some of them had large gaps, indicating that the corrosion product layer had become thicker. After 4 days of immersion, the corrosion products deposited on the surfaces of all specimens further increased, with nearly spherical corrosion products accumulating more densely, particularly on the surface of the Ca_2_Mg_6_Zn_3_ specimen. However, some areas on the surface of the MgZn_2_ specimen may still be uncovered by corrosion products. For the CaZn_13_ and Ca_2_Mg_6_Zn_3_ specimens, cracked corrosion product layers were also observed in the relatively flat pits between the clusters of corrosion product particles, indicating that the surfaces of the two specimens were all covered with corrosion products. After 7 days of immersion tests, all specimen surfaces were covered with corrosion products. Due to the limited growth space, some corrosion product particles came into contact with each other and squeezed together during the growth process, forming larger corrosion product particles.

**Figure 13:**
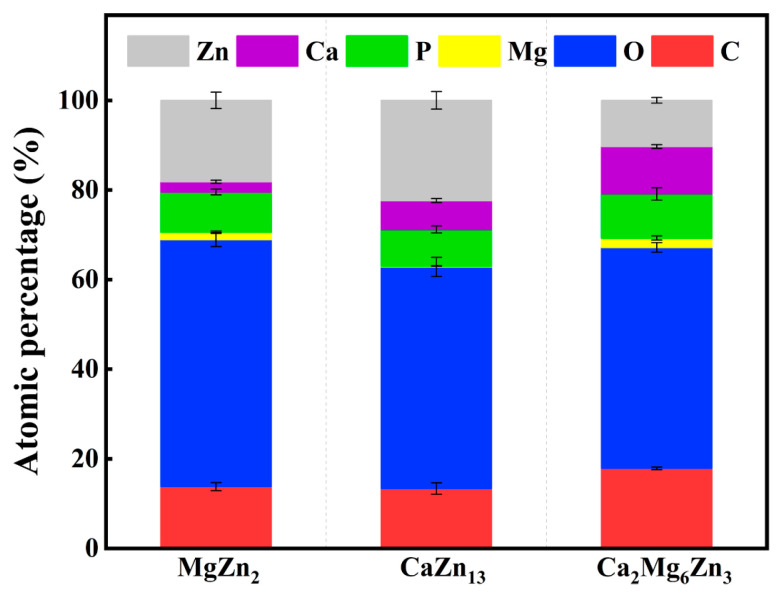
EDS results of the intermetallic compounds after immersion in SBF for 7 days.

The EDS results of the corrosion products on the specimens after 7 days of immersion are shown in Figure 13. It can be seen that all the corrosion products contain the elements Zn, Ca, P, Mg, O, and C, which is consistent with the EDS results of the corrosion products deposited on Zn-based implants after immersion tests in Hank’s solution or SBF for 28 days, as reported by Yuan et al. [36] and Zhang et al. [3]. The MgZn_2_ specimen does not contain Ca; therefore, the detected Ca element on this specimen evidently originates from the SBF solution. Similarly, the Mg element present on the CaZn_13_ surface also comes from the immersion solution. In addition, the Mg content on the MgZn_2_ specimen is higher than that on the CaZn_13_ specimen (1.67% vs. 0.20%); the additional Mg is likely derived from the degradation of the MgZn_2_ itself. Likewise, the extra Ca detected on the CaZn_13_ specimen is likely a result of the degradation of the CaZn_13_ itself. Based on this analysis, it can be further inferred that the higher contents of Ca and Mg on the Ca_2_Mg_6_Zn_3_ specimen are partly due to its own degradation. Among the three intermetallic compounds, the Ca_2_Mg_6_Zn_3_ specimen has the fastest degradation rate (Table 2) and the highest pH value during the immersion tests (Figure 11). The fastest degradation rate means that more Mg^2+^ and Ca^2+^ could be accumulated on the Ca_2_Mg_6_Zn_3_ specimen after immersion in SBF for the same time. The higher pH value was beneficial for the deposition of calcium phosphates on the specimen [39].

**Figure 14:**
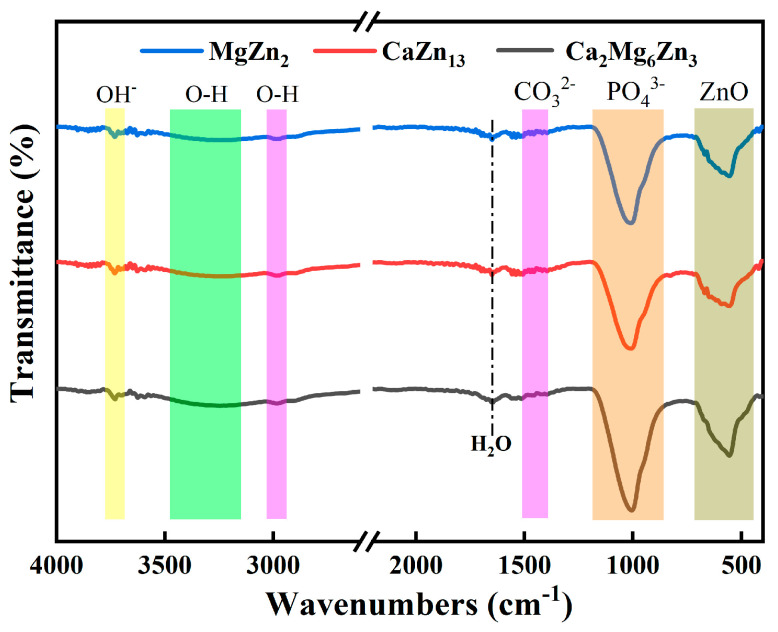
FTIR spectra of the intermetallic compounds after immersion in SBF for 7 days.

The FTIR spectra of the intermetallic compounds MgZn_2_, CaZn_13_, and Ca_2_Mg_6_Zn_3_ specimens after immersion in SBF for 7 days are shown in Figure 14. It can be seen that the corrosion products deposited on the three compounds have similar FTIR spectrums. The characteristic peaks of the functional groups in the FTIR spectra indicate that the corrosion products deposited on the compounds contain ZnO (553 cm^−1^) [40,41,42], PO_4_^3−^ (1006 cm^−1^) [40,41,42,43], CO_3_^2−^ (1400–1500 cm^−1^) [40,41,42,43], absorbed water (3100–3500 cm^−1^, 2980 cm^−1^, and 1651 cm^−1^) [43,44,45], and OH^−^ (3735 cm^−1^) [44,45]. The results are also similar to the FTIR results of the corrosion products deposited on Zn-based implants after immersion tests in Hank’s solution or SBF for 28 days, as reported by Yuan et al. [36] and Zhang et al. [3].

Generally, Zn(OH)_2_ and ZnO are the most common corrosion products when Zn-based implants degrade in vitro or in vivo [1,3,35,36,46,47,48,49]. The involved anodic reaction for the degradation of Zn is described in Equation (5), while the corresponding cathodic reaction is shown in Equation (4). The Zn^2+^ produced in Equation (5) reacts with the OH^−^ generated in Equation (4) to form Zn(OH)_2_ (Equation (6)). This Zn(OH)_2_ can subsequently be converted into the more stable ZnO, as represented in Equation (7), with prolonged immersion or implantation time. Some references have also reported corrosion products caused by the degradation of MgZn_2_ and Mg_2_Zn_11_ in corrosive media. For example, Byun et al. [32] reported that Zn(OH)_2_ was found on the surfaces of MgZn_2_ and Mg_2_Zn_11_ specimens that had undergone electrochemical tests in 3.5% NaCl solution. Besides that, MgO was also detected on the MgZn_2_ surface.

For the intermetallic compounds prepared in this work, their anodic degradation reactions can be expressed by Equations (1)–(3), respectively. Since all intermetallic compounds can generate Zn^2+^ during the degradation process, it is reasonable to infer, based on the experimental results of degradation products of numerous Zn-based implant materials [1,3,35,36,46,47,48,49], the report by Byun et al. [32], and the results shown in Figure 13 and Figure 14, that Zn(OH)_2_ and ZnO are likely present in the corrosion products deposited on the three intermetallic compounds after the immersion tests. Additionally, the Mg^2+^ produced by the degradation of the intermetallic compounds may also be converted into MgO or other compounds containing Mg element. It is known that the cations accumulated on the surfaces of the compounds can absorb anions [35], such as PO_4_^3−^ and CO_3_^3−^, present in the immersion solutions, and the degradation products generally contain various phosphates and carbonates containing Zn or Ca [3,23,35,36,46]. For example, Yuan et al. [36] and Zhang et al. [3] reported that the corrosion products deposited on the PLA/Zn composite and porous Zn-Mg-Y scaffolds after immersion in Hank’s solution or SBF for 28 days contain zinc phosphate and calcium carbonate. Due to the similar EDS and FTIR results of the corrosion products deposited on the intermetallic compounds in this work as in references [3,36], it can be reasonably inferred that the corrosion products deposited on the compounds should also contain zinc phosphate and calcium carbonate in addition to Zn(OH)_2_ and ZnO. Of course, further experiments are needed to verify the inference.

Zn = Zn^2+^ + 2e^−^ (5)

Zn^2+^ + 2OH^−^ = Zn(OH)_2_ (6)

Zn(OH)_2_ = ZnO + H_2_O (7)

**Figure 15:**
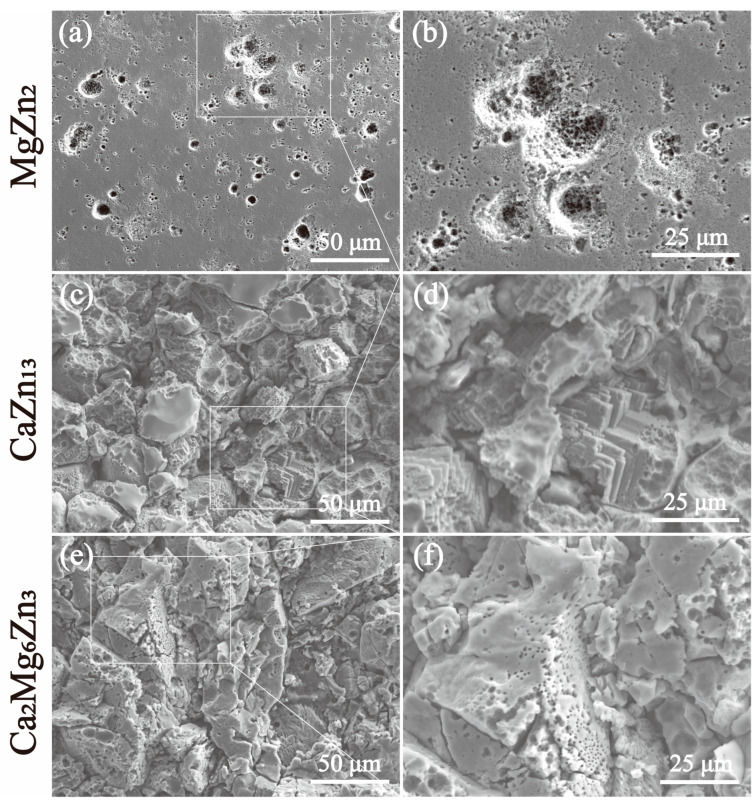
SEM images of the MgZn_2_ (**a**,**b**), CaZn_13_ (**c**,**d**) and Ca_2_Mg_6_Zn_3_ (**e**,**f**) specimens after immersion in SBF for 7 days and subsequent removal of the corrosion products with a chromic acid solution.

The SEM images of the intermetallic compounds after immersion in SBF for 7 days, followed by cleaning of the corrosion products with a chromic acid solution, are shown in Figure 15. During the 7-day immersion test, severe localized corrosion occurred on the surface of the MgZn_2_ specimen, resulting in an uneven distribution of corrosion pits with different sizes (Figure 15a). Even the areas that appear flat at low magnification (Figure 15a) also contain many small corrosion pits when viewed at high magnification (Figure 15b). For the CaZn_13_ and Ca_2_Mg_6_Zn_3_ specimens, the original flat surfaces were no longer visible; instead, the surfaces became very rough and uneven, indicating that severe corrosion occurred during the immersion tests. Especially for the Ca_2_Mg_6_Zn_3_ specimen, the remaining exposed surface is riddled with holes, indicating that this specimen experienced the most severe corrosion (Figure 15e,f). Clearly, the degrees of corrosion for the specimens are consistent with the degradation rates of the compounds presented in Table 2.

### 3.7. Cytotoxicity of the Intermetallic Compounds

**Figure 16:**
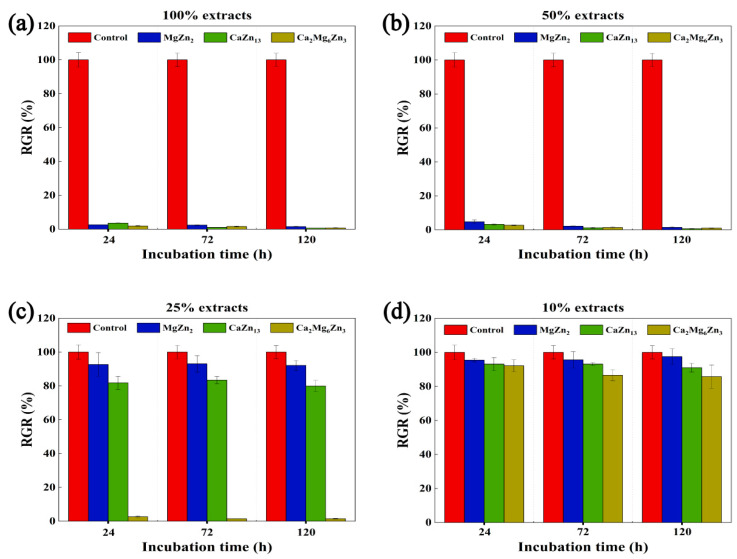
The RGRs of MC3T3-E1 cells cultured in the extracts with (**a**) 100%, (**b**) 50%, (**c**) 25%, and (**d**) 10% concentrations for 24–120 h.

Figure 16 shows the RGRs of MC3T3-E1 cells cultured in the extracts at concentrations of 100%, 50%, 25%, and 10% for 24 to 120 h. It is evident that both the 100% and 50% extracts of all specimens exhibited severe cytotoxicity on MC3T3-E1 cells regardless of whether the cells were cultured for 24, 72, or 120 h (grade 4 or 5). When the extract concentrations were reduced to 25%, except the extract prepared from the Ca_2_Mg_6_Zn_3_ specimen, the other two extracts showed good cellular activity (grade 0 or 1). Only when the extracts were further diluted to 10%, as the other two extracts demonstrated, did the extract prepared from the Ca_2_Mg_6_Zn_3_ specimen also show good cellular activity on MC3T3-E1 cells.

The measured Zn^2+^ concentrations in the 100% extracts prepared from the MgZn_2_, CaZn_13_, and Ca_2_Mg_6_Zn_3_ specimens are 49.8, 56.6, and 86.7 μg/mL, respectively. These concentrations are all greater than that measured in the 100% extract of a pure Zn specimen (approximately 16.54 μg/mL [50] or 18.98 μg/mL [20]). Clearly, these Zn^2+^ concentrations are roughly proportional to the degradation rates of the corresponding compounds and the pure Zn specimen, as indicated by the polarization curves (Table 2). The corrosion severity of the compounds after immersion in SBF (Figure 15) further verify the conclusion. It should be noted that although the Zn contents in MgZn_2_, CaZn_13_, and Ca_2_Mg_6_Zn_3_ compounds are different, the released Zn^2+^ ions during the degradation process still mainly depend on the degradation rates of the compounds rather than the Zn contents in the compounds. In addition to Zn^2+^, Mg^2+^ (21.8 μg/mL) and Ca^2+^ (9.4 μg/mL) were also detected in the 100% extracts of the MgZn_2_ and CaZn_13_ specimens, respectively. Meanwhile, Ca^2+^ (39.3 μg/mL) and Mg^2+^ (96.0 μg/mL) were found in the extract of the Ca_2_Mg_6_Zn_13_ specimen.

It is known that the in vitro cytotoxicity of Zn-based implants primarily depends on the Zn^2+^ concentration in the extracts. Since Zn is only a trace element in the human body (approximately 3 g) [27], the threshold concentration at which Zn^2+^ causes severe cytotoxicity on cells and tissues is quite low. According to the findings of Yang et al. [28], Yuan et al. [36], and Zhang et al. [3], the threshold concentration of Zn^2+^ that caused severe cytotoxicity on MC3T3-E1 cells was around 16.3 μg/mL. Based on this threshold, the 100% extracts prepared from the intermetallic compound specimens inevitably resulted in severe cytotoxicity on MC3T3-E1 cells. Even when the extracts were diluted to 50%, the concentrations of Zn^2+^ in the extracts of the MgZn_2_, CaZn_13_, and Ca_2_Mg_6_Zn_3_ specimens remained at 24.90, 28.30, and 43.35 μg/mL, respectively. Consequently, these extracts continued to exhibit severe cytotoxicity on MC3T3-E1 cells. Upon further dilution to 25%, the Zn^2+^ concentrations in the extracts of the MgZn_2_ and CaZn_13_ specimens decreased to below 16.3 μg/mL (12.45 and 14.15 μg/mL, respectively). As a result, these extracts showed good cellular activity. In contrast, the Zn^2+^ concentration in the 25% Ca_2_Mg_6_Zn_3_ extract remained higher than 16.3 μg/mL (21.68 μg/mL), which explains why this extract still exhibited severe toxicity on MC3T3-E1 cells. When the extracts were diluted to 10%, the Zn^2+^ concentrations in all extracts fell well below 16.3 μg/mL, resulting in all of these extracts showing excellent cellular activity.

Although Mg^2+^ and/or Ca^2+^ were also present in the extracts of the intermetallic compounds, the experimental results presented in this work indicated that they had little effect on the cytotoxicity of the extracts. This result may be related to the fact that Mg and Ca are major elements in the human body, with relatively high contents in the body (25 g and 1200 g, respectively) [27]. Therefore, higher ion concentrations may be necessary for Mg^2+^ and Ca^2+^ to cause severe cytotoxicity on MC3T3-E1 cells. In addition, the positive effect of Mg^2+^ and/or Ca^2+^ in the extracts on the cell viability remains unclear, and further experiments are needed in the future.

## 4. Conclusions

The intermetallic compounds MgZn_2_, CaZn_13_, and Ca_2_Mg_6_Zn_3_ were successfully prepared by a vacuum induction melting method. The phase structures, microstructures, and relevant properties of the intermetallic compounds were characterized. The main conclusions are as follows:

1. Both the annealed MgZn_2_ and CaZn_13_ specimens have high purity, while the annealed Ca_2_Mg_6_Zn_3_ specimen contains not only Ca_2_Mg_6_Zn_3_ phase but also Mg_2_Ca phase. After purifying the annealed Ca_2_Mg_6_Zn_3_ specimen (soaking in NaCl solution for some time), high purity Ca_2_Mg_6_Zn_3_ phase was obtained.
2. The MgZn_2_ and CaZn_13_ specimens exhibited good thermal stability below 773 K. However, the Ca_2_Mg_6_Zn_3_ specimen melted between 719.7 and 750.9 K.
3. The three intermetallic compounds were degraded according to the atomic ratio of the elements in each compound in corrosive media.
4. The corrosion potentials of the three intermetallic compounds are all lower than that of pure Zn, that is, the three compounds can act as sacrificial anodes in Zn-matrix composites. Among the three compounds, MgZn_2_ has the highest corrosion potential (−1.063 V_SCE_ in SBF or −1.170 V_SCE_ in 0.9% NaCl solution), and Ca_2_Mg_6_Zn_3_ exhibits the lowest corrosion potential (−1.432 V_SCE_ in SBF or −1.490 V_SCE_ in 0.9% NaCl solution).
5. The degradation rates of the three intermetallic compounds in corrosive media are all greater than that of pure Zn. Among the three compounds, MgZn_2_ has the lowest corrosion current density (12.03 µA/cm^2^ in SBF or 4.37 µA/cm^2^ in 0.9% NaCl solution), and Ca_2_Mg_6_Zn_3_ exhibits the highest corrosion current density (66.23 µA/cm^2^ in SBF or 69.49 µA/cm^2^ in 0.9% NaCl solution). After immersion in SBF for 7 days, localized corrosion occurred on the MgZn_2_. CaZn_13_ and Ca_2_Mg_6_Zn_3_ experienced severe corrosion during 7 days of immersion tests.
6. The rapid degradation of the three compounds led to greater Zn^2+^ dissolution, resulting in severe cytotoxicity. But when the extracts were diluted to 10%, all extracts exhibited good cell activity.

## Figures and Tables

**Table 1:** Chemical composition of the used SBF [35].

Number	Reagent	Concentration (g/L)
1	NaCl	8.035
2	NaHCO_3_	0.355
3	KCl	0.225
4	K_2_HPO_4_∙3H_2_O	0.231
5	MgCl_2_∙6H_2_O	0.311
6	CaCl_2_	0.292
7	Na_2_SO_4_	0.072

**Table 2:** Electrochemical parameters obtained from the polarization curves shown in Figure 10.

Specimens	SBF		0.9% NaCl	
	*E*_corr_ (V_SCE_)	*I*_corr_ (µA/cm^2^)	*E*_corr_ (V_SCE_)	*I*_corr_ (µA/cm^2^)
Pure Zn	−1.003	5.84	−1.034	3.53
MgZn_2_	−1.063	12.03	−1.170	4.37
CaZn_13_	−1.289	12.93	−1.311	31.66
Ca_2_Mg_6_Zn_3_	−1.432	66.23	−1.490	69.49

**Table 3:** The metal ion concentrations in 0.9% NaCl solutions after soaking the intermetallic compounds for 24 h.

Specimens	Metal Ion Concentration (mmol/L)			Concentration
	Ca^2+^	Mg^2+^	Zn^2+^	Ratio
MgZn_2_		34.81 ± 1.52	65.19 ± 1.50	Mg^2+^/Zn^2+^ = 1/1.87
CaZn_13_	7.00 ± 1.67		93.00 ± 1.67	Ca^2+^/Zn^2+^ = 1/13.29
Ca_2_Mg_6_Zn_3_	17.63 ± 1.02	54.08 ± 1.24	28.29 ± 0.26	Ca^2+^/Mg^2+^/Zn^2+^ = 1/3.07/1.60

## Data Availability

The original contributions presented in this study are included in the article/supplementary material. Further inquiries can be directed to the corresponding authors.
